# Anatomical study of simple landmarks for guiding the quick access to humeral circumflex arteries

**DOI:** 10.1186/1471-2482-14-39

**Published:** 2014-06-26

**Authors:** Yi-xin Chen, Yi Zhu, Fu-hua Wu, Xin Zheng, Yu-fan Wangyang, Han Yuan, Xiao-xiao Xie, Dong-ya Li, Chang-jun Wang, Hong-fei Shi

**Affiliations:** 1 Department of Orthopaedics, Nanjing Drum Tower Hospital, The Affiliated Hospital of Nanjing University Medical School, No. 321 Zhongshan Road, Nanjing China; 2 Department of Orthopedics, Lishui County People’s Hospital of Nanjing, Nanjing, China

**Keywords:** Axillary artery, Circumflex humeral artery, Proximal humeral fracture

## Abstract

**Background:**

The posterior and anterior circumflex humeral artery (PCHA and ACHA) are crucial for the blood supply of humeral head. We aimed to identify simple landmarks for guiding the quick access to PCHA and ACHA, which might help to protect the arteries during the surgical management of proximal humeral fractures.

**Methods:**

Twenty fresh cadavers were dissected to measure the distances from the origins of PCHA and ACHA to the landmarks (the acromion, the coracoid, the infraglenoid tubercle, the midclavicular line) using Vernier calipers.

**Results:**

The mean distances from the origin of PCHA to the infraglenoid tubercle, the coracoid, the acromion and the midclavicular line were 27.7 mm, 50.2 mm, 68.4 mm and 75.8 mm. The mean distances from the origin of ACHA to the above landmarks were 26.9 mm, 49.2 mm, 67.0 mm and 74.9 mm.

**Conclusion:**

Our study provided a practical method for the intraoperative identification as well as quick access of PCHA and ACHA based on a series of anatomical measurements.

## Background

With an aging population, proximal humeral fracture, the third most common fracture in elderly patients, presents an increasing challenge for the healthcare system. Although the majority of the fractures can be treated conservatively, surgical reduction and internal fixation is recommended when the fracture is displaced and unstable [1,2]. Among the postoperative complications, avascular necrosis (AVN) of the humeral head is not uncommon, which may lead to partial or total collapse of the humeral head. The unfavorable subsequence includes functional impairment, pain, varus malunion of the humeral head, and increased risk of screw perforation [3]. It is important to avoid iatrogenic damage to the blood supply of humeral head during surgery, and hopefully reduce the incidence of AVN.

The posterior and anterior circumflex humeral artery (PCHA and ACHA) are proven crucial for the blood supply of humeral head [4]. Both of the arteries typically originate from the third part of axillary artery, run in close proximity to the proximal humerus, and are prone to damage in the settings of proximal humeral fractures and related surgeries (e.g. open reduction and internal fixation of the fracture, shoulder arthroplasty, etc.) [5-8]. Previously, most of the studies of the PCHA and ACHA mainly focused on their contributions to the blood supply of humeral head [4,9-11]. Meanwhile, the clinical guideline is lacking, considering how to avoid iatrogenic damage to the PCHA and ACHA during surgery. Besides, the anatomical characteristics of the circumflex arteries need to be further addressed when the repair and reconstruction of the arteries are required. In the present study, we aimed to identify simple anatomical landmarks for guiding the quick access to PCHA and ACHA in cadavers, which might provide useful information in protecting and even repairing the arteries during the surgical management of proximal humeral fractures.

## Methods

The protocol of this study was approved by the Committee on Medical Ethics of Nanjing Drum Tower Hospital (Ref. No. 110309). Dissection was performed in 20 donated bodies to science, which included 12 male and 8 female with an average age of 66.2 years (ranged from 32 to 83 years). In each body, both of the left and right shoulders were carefully dissected. The pectoralis major and pectoralis minor were cut from their insertions and retracted medially. The origin of the coracobrachialis and short head of the biceps brachialis were detached from the coracoid process and retracted distally to expose the axillary artery and brachial plexus. The PCHA and ACHA were identified and carefully dissected along the axillary artery. The PCHA was considered classic if it arose from the third part of the axillary artery and traveled posteriorly around the proximal humerus. The ACHA was defined as classic if it arose from the third part of the axillary artery and followed a horizontal course to the bicipital groove [12,13].

The following measurements were performed using Vernier calipers with the arm abducted to 60 degrees in supine position: 1) the distances from the origins of PCHA and ACHA to the infraglenoid tubercle (PI and AI), the anterior-inferior border of the coracoid process (PC and AC), the lateral border of the acromion (PA and AA), and the midclavicular line (PM and AM); 2) the distances from the origins of PCHA and ACHA to their first branches. The distances measured form the left and right shoulder of the same body were averaged to represent the final measurements.

All the quantitative data were presented as mean ± SD. The distances from the origins of PCHA and ACHA to the landmarks were analyzed using paired t test to detect the difference between the left and right shoulder. The SPSS version 15.0 software (SPSS Inc, Chicago, IL, USA) was used and statistical significance was accepted at *P* < 0.05.

## Results

### The origins of PCHA and ACHA

**Figure 1:**
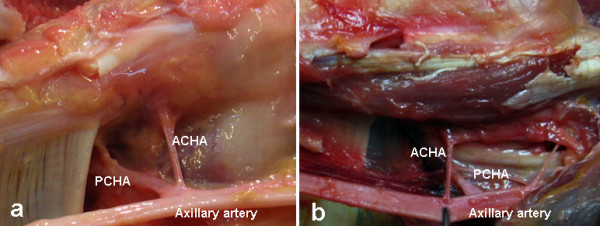
The PCHA and ACHA were found originated from the third part of the axillary artery in a common origin (a) or in separated origins (b).

Among the 20 bodies, the bilateral ACHA and PCHA were found symmetric in 19 bodies. Only one case showed un-symmetric arteries with the right ACHA originated from the axillary artery and shared a common origin with the PCHA whereas the left ACHA was absent. The PCHA branched from the axillary artery in 36 of the 40 shoulders (90%), originated from the deep brachial artery in 2 shoulders and from the subscapular artery in 2 shoulders. The ACHA originated from the axillary artery in 39 of the 40 shoulders (98%) and shared the common origin with the PCHA in 13 of the 40 shoulders (33%) (Figure 1).

The mean distance from the origin to the first division of the PCHA was 47.1 mm (31 to 76 mm).The mean distance from the origin to the first division of the ACHA was 25.8 mm (18 to 35 mm). Comparing the distance between the right and the left sides, no significant difference was observed.

### The distances from the origins of PCHA and ACHA to the bony landmarks

The mean distances from the origin of the PCHA to the infraglenoid tubercle (PI), the coracoid (PC), the acromion (PA) and the midclavicular line (PM) were 27.7 mm (18 to 38 mm), 50.2 mm (41 to 62 mm), 68.4 mm (60 to 79 mm) and 75.8 mm (70 to 83 mm) respectively. The mean distances from the origin of the ACHA to the infraglenoid tubercle (AI), the coracoid (AC), the acromion (AA) and the midclavicular line (AM) were 26.9 mm (20 to 39 mm), 49.2 mm (45 to 62 mm), 67.0 mm (64 to 80 mm) and 74.9 mm (71–85 mm) respectively. When the data from the right and the left side were compared, no significant difference was observed (Table 1). Apart from these measurements, the origins of the PCHA and ACHA were observed distal and lateral to the coracoid process, distal and medial to the acromion, distal and lateral to the infraglenoid tubercle, and lateral to the midclavicular line in all of the donated bodies.

**Table 1:** The measured distances from the origins of PCHA and ACHA to the landmarks

	**PI (mm)**	**AI (mm)**	**PC (mm)**	**AC (mm)**	**PA (mm)**	**AA (mm)**	**PM (mm)**	**AM (mm)**
Left side	28.1 ± 6.2	26.4 ± 6.2	50.3 ± 5.3	48.3 ± 5.9	69.3 ± 3.3	67.4 ± 4.1	75.8 ± 5.8	74.9 ± 6.1
Right side	27.2 ± 6.2	27.4 ± 7.0	50.1 ± 5.7	50.0 ± 6.3	67.5 ± 5.9	66.5 ± 5.3	75.8 ± 6.3	74.8 ± 6.1
*P* value	0.724	0.749	0.924	0.536	0.423	0.699	0.993	0.979

Data were presented as means ± SD.

**Figure 2:**
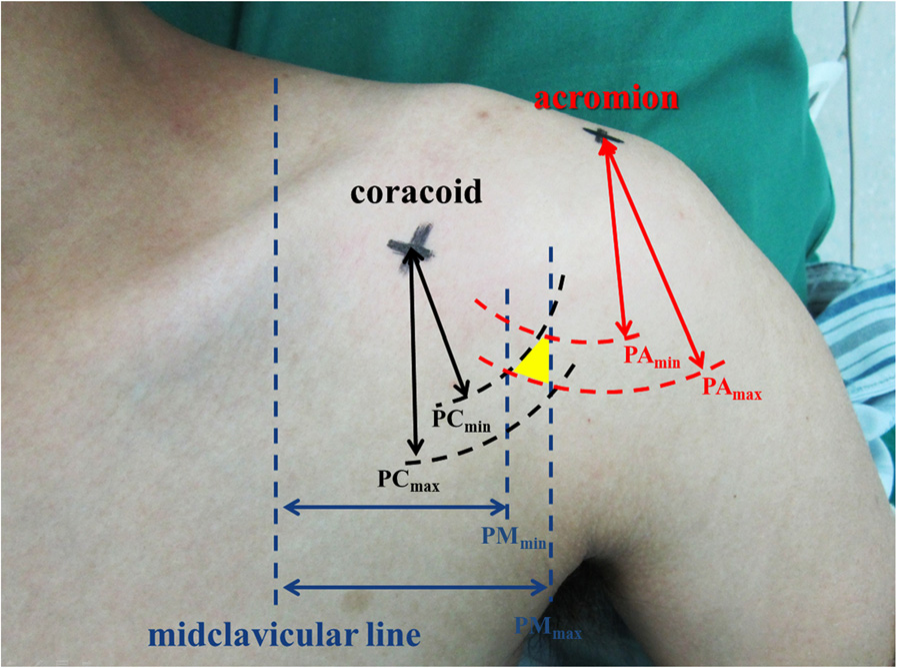
**Quick access to the origins of the PCHA and ACHA.** The landmarks including the acromion, the coracoid, and the midclavicular line were identified on the body surface. According to our measurements, the origin of the PCHA and ACHA were located within the overlapping area highlighted in yellow color.

According to these data, the origins of the PCHA and ACHA from the axillary artery could be located intraoperatively based on the landmarks. In brief, the acromion, the coracoid, and the midclavicular line were identified on the body surface. Two vertical lines were drawn with a distance of PM_min_ (the minimum value of PM) and PM_max_ (the maximum value of PM) to the midclavicular line respectively. Two arcs centered to the coracoid were drawn with a radius of PC_min_ and PC_max_ respectively. Another two arcs centered to the acromion were also drawn with a radius of PA_min_ and PA_max_ respectively. The origin of the PCHA were then located within the overlapping area formed by the six lines (Figure 2). The origin of the ACHA could be identified using similar procedure.

## Discussion

During the surgical management of proximal humeral fractures, careful dissection of the soft tissue and gentle manipulation of the fracture were considered important to protect blood supply. In the current study, we investigated the anatomical characteristics of the PCHA and ACHA, the key vessels for the blood supply of humeral head, and developed a method for quick access to the arteries according the bony landmarks. This study is of clinical relevance as it helps to avoid potential iatrogenic damage to the circumflex humeral arteries. Besides, the quick location of the origins of the circumflex humeral arteries is extremely important for hemostasis and subsequent repair of the vessels.

Considering the origin of the circumflex arteries, Hettrich reported that both of the PCHA and ACHA branched from the axillary artery, while PCHA originated from the common trunk with ACHA in 29% of the specimens [4]. In another study, Duparc observed that PCHA originated from the axillary artery in 29 of 32 adult shoulders (91%) and shared common origin with ACHA in 10 cases (31%) [14]. Comparatively, our study showed that 98% of the ACHA and 90% of the PCHA originated from the axillary artery, while 33% of the PCHA shared same origin with ACHA. Also, the PCHAs were found to originate from the deep brachial artery in two specimens, and from the subscapular artery in two shoulders. These variations were consistent with Olinger’s report wherein the PCHA was observed to originate from the deep brachial artery, the subscapular artery, or the lateral thoracic artery [13].

Between the two circumflex arteries, ACHA was previously reported to gives off one arcuate artery which entered the proximal humerus to provide dominant blood supply for the humeral head [9,10]. However, PCHA was believed to play a greater role in the blood supply to humeral head in most of the recent studies [4,11,14]. In our study, the diameter of PCHA was observed to be much larger than that of ACHA, which provided indirect evidence for the dominance of PCHA in the blood supply of humeral head.

Despite of the relatively low incidence, intraoperative injury of PCHA or ACHA was of clinical importance. Undiagnosed injury of PCHA could be life-threatening [6]. The injured PCHA and ACHA were also reported to cause serious stenosis of axillary artery and lead to impaired shoulder function [15]. When the PCHA or ACHA was injured unexpectedly, prompt location of the origins of PCHA and ACHA would be significantly valuable. As shown in our study (Figure 2), the midclavicular line and the bony landmarks like the acromion, the coracoid, and the infraglenoid tubercle could be used to locate the origins of PCHA and ACHA. If the classic deltopectoral approach was used, the origins of PCHA and ACHA could be quickly expose through blunt dissection of the pectoralis major. Via compressing the origins of the arteries, the massive hemorrhage could usually be controlled temporarily. Decisions could then be made to ligate or to repair the arteries. Because the circumflex humeral arteries are crucial for the blood supply of humeral head, the authors tend to repair instead of ligate the vessels in case of bleeding. The quick location of the origins of the vessels will benefit the vessel graft procedure when direct anastomosis cannot be performed.

This study has several limitations. Since the position of the axillary artery is mobile during motion of shoulder joint, the PCHA and ACHA can be quickly located using our method only when the arm is abducted to 60 degrees in supine position. Besides, although the bony landmarks were easily identified in all the cadavers used in this study, an obese patient wound theoretically increase the difficulty and lower the accuracy of our methods. Third, we only analyzed the anatomical characteristics of the origins of the PCHA and ACHA in this study, while further studies would be conducted to analyze the travel, distribution and variation of the circumflex arteries lateral to their origins.

## Conclusions

In conclusion, this study provided a practical method for the intraoperative identification as well as quick access of PCHA and ACHA based on a series of anatomical measurements.

## Competing interests

The authors declare they have no conflict of interest.

## Authors’ contributions

All authors read and agreed with the contents of the manuscript. YXC and HFS participated in study design and manuscript drafting. YZ, FHW, and XZ participated in cadaver experiment. YFW, HY, and XXX performed data collection. DYL and CJW helped in statistical analysis. All authors read and approved the final manuscript.

## Pre-publication history

The pre-publication history for this paper can be accessed here:

http://www.biomedcentral.com/1471-2482/14/39/prepub
